# A cost-utility analysis of the transcutaneous Osia System® compared to the percutaneous bone-conduction hearing implant Baha® Connect for hearing loss in Spain

**DOI:** 10.3389/fpubh.2026.1829029

**Published:** 2026-06-24

**Authors:** Serafín Sánchez-Gómez, Rubén Polo, Francisco Ibáñez-Ortigosa, Óscar Martínez-Pérez, Víctor Zamora, Jesús Cuervo

**Affiliations:** 1 Department of Otolaryngology and Head and Neck Surgery, Hospital Universitario Virgen Macarena, Sevilla, Spain; 2 Department of Otolaryngology, Hospital Universitario Ramón y Cajal, Madrid, Spain; 3 Axentiva Solutions S.L., Barcelona, Spain; 4 Axentiva Solutions S.L., Oviedo, Spain

**Keywords:** bone conduction hearing implants, cost-utility analysis, health economics, hearing loss, Osia® system, Spain

## Abstract

**Objective:**

To evaluate the cost-effectiveness of the transcutaneous bone-conduction hearing implant Osia® System compared to the percutaneous bone-conduction hearing implant Baha® Connect System in a pooled population of adults eligible for bone-conduction hearing implants, from the perspective of the Spanish National Health System.

**Methods:**

A lifetime Markov model with 6-month cycles was simulated based on a previously published Swedish study, incorporating three primary health states: “with implant,” “without implant,” and “dead.” Clinical inputs included probabilities of adverse events (e.g., complications, device failure), health utilities in Quality-Adjusted Life Years (QALYs), and direct healthcare costs. Costs and QALYs were discounted at a 3% annual rate. Deterministic and probabilistic sensitivity analysis were conducted to assess the uncertainty of the model parameters.

**Results:**

In the base-case analysis, the Osia® System incurred a total cost of €24,555 per patient and yielded 15.94144 QALYs, while the Baha® Connect System accounted for €20,968 and generated 15.17033 QALYs. The incremental cost-effectiveness ratio (ICER) of the Osia® System was €4,651/QALY gained, compared to the Baha® Connect System. The model was most sensitive to utility values for each device. At a willingness-to-pay threshold of €30,000/QALY, the probabilistic sensitivity analysis showed that the Osia® System had a 62% probability of being cost-effective.

**Conclusion:**

From the Spanish healthcare system perspective, the Osia® System is a cost-effective alternative to the Baha® Connect System for the overall adult patient population with hearing loss in Spain eligible for a bone-conduction hearing implant. These results support its inclusion in national coverage and reimbursement policies to improve clinical and patient-reported outcomes and to promote sustainable healthcare delivery. However, these findings should be interpreted cautiously due to the reliance on non-Spanish clinical inputs and the absence of subgroup-specific analyses.

## 1 Introduction

Hearing loss constitutes a significant global public health challenge, with profound implications for individuals’ quality of life, mental health, and socioeconomic participation, and an estimated global annual economic burden of nearly one trillion United States dollars (1). This burden could be particularly acute within the context of a rapidly aging population, as the prevalence of hearing loss increases dramatically with age (2). According to the 2017 Spanish National Health Survey, 44.1% of individuals over 64 reported hearing difficulties, placing auditory impairment among the top three chronic issues in older adults and underscoring its significant public health impact (3). Additionally, untreated hearing loss frequently leads to social isolation, loneliness, and increased dependency (4), and is recognized as a risk factor for accelerated cognitive decline (5), thereby placing a substantial and growing strain on patients, their families, and the national healthcare system.

For a specific cohort of patients—those with conductive hearing loss (CHL), mixed hearing loss (MHL), or single-sided deafness (SSD)—conventional hearing aids are often clinically unsuitable or ineffective due to anatomical or functional limitations. This heterogeneous group is eligible for bone-conduction hearing implants (BCHIs), which have become the established standard of care, offering effective auditory rehabilitation by directly stimulating the cochlea through bone-conducted mechanical vibrations produced by the implant (6). Notably, one of the most effective and widely adopted solutions in the Spanish National Health System (SNS) for many years has been the Baha® Connect System, a percutaneous BCHI. This system functions via a small titanium abutment surgically implanted into the temporal bone behind the ear and protruding through the skin. This design creates a direct, physical connection to an external sound processor, maximizing the transfer of sound vibrations to the inner ear (7).

While audiologically effective, the Baha® Connect System’s defining feature—its percutaneous skin-penetrating abutment—is also its most significant drawback. This design carries a potential risk of cutaneous complications. Patients might experience adverse events at the abutment site, including chronic skin irritation and recurrent infections, which sometimes require hospitalization (8). This problem has been addressed in Spanish clinical practice, with a long-term observational study from a tertiary hospital reporting postoperative complications in 30.5% of patients with percutaneous implants (9). Clinical practice is mainly guided by a Spanish Society of Otolaryngology and Head and Neck Surgery (SEORL-CCC) consensus document (6), as the SNS has not yet issued an official Clinical Practice Guideline for BCHIs.

In response to the well-documented limitations of percutaneous devices, the Osia® System was developed as a next-generation, active transcutaneous BCHI. Unlike the Baha® Connect System, the Osia® System is entirely subcutaneous, eliminating the need for a skin-penetrating abutment and thereby substantially reducing the risk of soft tissue complications (10). Clinical evidence from a prospective, multi-center international trial has demonstrated that the Osia® System is not only safe but also provides significant and clinically meaningful improvements in hearing thresholds, speech perception in noisy environments, and overall health-related quality of life (11). By combining direct bone conduction stimulation with a transcutaneous design, the Osia® System offers the audiological benefits of percutaneous implants while effectively mitigating their known complications, representing a substantial advancement in patient outcomes.

The SNS is founded on principles of universality, equity, and financial sustainability of healthcare provision. To ensure that coverage and reimbursement decisions are evidence-based, Health Technology Assessment (HTA) plays a key role within the SNS framework (12). In accordance with the standards set by the GANT Guides (New Technology Acquisition Guide), an economic evaluation must be performed to comprehensively assess a medical device and inform decision-making. (13). With the recent implementation of the EU Regulation 2021/2282 on HTA, Spain is aligning its assessment processes with European standards, including joint clinical assessments of high-risk medical devices. Despite the widespread use of BCHIs, no formal appraisal exists for the Osia® System or other BCHIs in Spain, which represents a critical evidence gap for clinicians and policymakers.

Therefore, the objective of this analysis is to evaluate the cost-effectiveness of the Osia® System compared to the established Baha® Connect System in a pooled population of adults eligible for BCHI (encompassing CHL, MHL, and SSD), from the perspective of the Spanish National Health System, providing the evidence needed to guide therapeutic positioning and reimbursement decisions.

## 2 Materials and methods

### 2.1 Literature review

A narrative literature review was conducted in MEDLINE and Google Scholar to identify key inputs for the Spanish setting. This targeted review focused on sourcing data and parameters necessary to adapt the proposed model to the Spanish landscape. The aim was to ensure the model’s assumptions and variables reflected the national context.

### 2.2 Model and scope

A model-based cost-utility analysis was conducted through the adaptation of a previously published analysis by Ghineli et al. (14) to the Spanish context. Although the effectiveness results included various percutaneous BCHI, the Baha® Connect System was considered the comparator in this economic evaluation due to the availability of specific cost data for the device.

**Figure 1:**
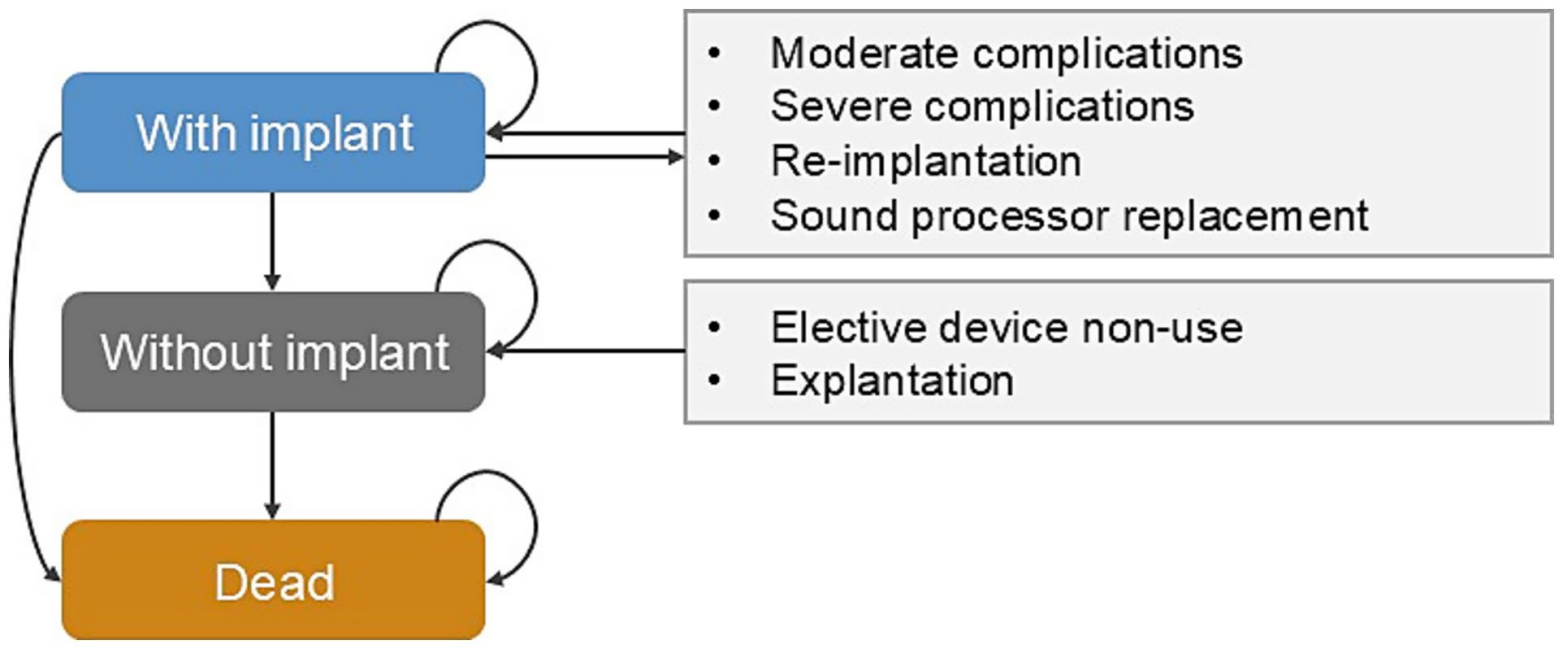
Model structure. It was adapted from the study of Ghinelli F et al. (14).

The Markov model included 6-month cycles, simulated the lifetime experience of a cohort of patients receiving either the Osia® System or the Baha® Connect System. The model consisted of three main health states (Figure 1):

1. *“With implant”*: Patients have a functioning BCHI (either Osia® or Baha®). Within this state, patients can experience device-related events such as moderate complications (requiring physician visits and conservative management), severe complications (requiring hospitalization or revision surgery), explantation (device removal, potentially followed by reimplantation), or routine sound processor replacement. All patients entered the model in this health state.
2. *“Without implant”*: Patients no longer use a BCHI. This state can be entered due to elective non-use (e.g., discontinuation due to perceived low effectiveness or burden) or following explantation without subsequent reimplantation (e.g., due to severe complications or patient choice). Patients in this state are assumed to incur no further direct costs or risks associated with the implant.
3. *“Dead”*: An absorbing state representing mortality from any cause.

A half-cycle correction was applied to costs and Quality-Adjusted Life Years (QALYs) to account for the timing of events within each cycle. In line with recommendations from Spanish guidelines for economic evaluations of health technologies (15), a 3% annual discount rate was applied to both costs and QALYs.

### 2.3 Base-case analysis

This analysis compared the Osia® System with the Baha® Connect System. The base-case analysis calculated the total lifetime costs, total lifetime QALYs, incremental costs, and incremental QALYs for the Osia® System compared with Baha® Connect System. The Incremental Cost-Effectiveness Ratio (ICER), representing the additional cost per QALY gained, was the main outcome measure.

### 2.4 Sensitivity analysis

One-way sensitivity analyses were conducted to assess the impact of uncertainty in individual model parameters on the ICER. All parameters were varied within ranges ±20% of the base-case value. The results of the deterministic sensitivity analysis (DSA) were presented using a tornado diagram to identify the most influential parameters.

A probabilistic sensitivity analysis (PSA) was also performed using Monte Carlo simulation, running 1,000 iterations of the model. In each iteration, parameter values were simultaneously drawn from predefined probability distributions. The results of the PSA were presented as a scatter plot on the cost-effectiveness plane and as a cost-effectiveness acceptability curve.

### 2.5 Clinical inputs

The model was populated with a cohort of patients aged 59 years and older with hearing loss up to 55 dB who were eligible for a BCHI. Since the pragmatic literature review did not identify any clinical inputs for the Spanish context that could fit in the model, international clinical inputs from the Swedish model were used as a reference (Table 1).

**Table 1:** Key model input parameters for the Spanish adaptation.

Devices	Complication	6 months probability	Sources
Osia® system	Moderate complication	1.35%	Key et al. (26)
	Severe complication	1.80%	Key et al. (26)
	Explantation^†^	6.10%	Key et al. (26)
	Reimplantation^‡^	78.04%	Crowder et al. (27)
	Without use	2.60%	Cowan et al. (28)
Baha® connect system	Moderate complication	2.72%	Teunissen et al. (29)
	Severe complication	1.93%	Teunissen et al. (29)
	Explantation^†^	10.12%	Teunissen et al. (29)
	Reimplantation^‡^	83.24%	Teunissen et al. (29)
	Without use	0.58%	Teunissen et al. (29)

^†^Severe complications dependent; ^‡^Explantation dependent.

Regarding Baha® Connect inputs, in the absence of clinical sources reporting results exclusively for Baha® Connect, the model utilizes data from studies providing a blended representation of percutaneous systems, assuming clinical equivalence for this device (8).

To reflect the Spanish context, transition probabilities to the “death” health state were based on local mortality data from the National Statistics Institute (INE) (17). Due to data scarcity, CHL, MHL, and SSD were modeled as a single pooled cohort, assuming clinical homogeneity. The transfer of international data is justified using standardized audiological criteria, surgical pathways and patient baseline similarities across European settings (17).

### 2.6 Economic inputs

Complications and procedure costs for the Spanish context were calculated by estimating the resources usage and applying the unit costs published in Valcárcel-Nazco et al. (18), who developed a comprehensive and standardized database of healthcare costs in Spain (CONCEPT-COSTS), designed to support economic evaluations and health services research. Unit costs were updated to 2026 euros by applying the Spanish General Consumer Price Index (CPI), based on official data from the INE (19). Detailed costs for complications, procedures, and devices are summarized in Table 2. All model assumptions underwent clinical validation to confirm their alignment with standard clinical practice in Spain and to ensure their conservative approach.

**Table 2:** Model costs.

Resource	Unit cost (2026)	Total cost
Moderate complications		€199.66
Otorhinolaryngology specialist visit	€147.43 (18)	
Nurse visit	€52.23 (18)	
Severe complications		€386.67
Otorhinolaryngology specialist visit	€147.43 (18)	
Nurse visit	€239.24 (18)	
Implantation/explantation		
Osia® system (major outpatient surgery)	€964.30 (18)	€964.30
Baha® connect (minor outpatient surgery)	€239.24 (18)	€239.24
Reimplantation		
Osia® system (major outpatient surgery + 35%)	€964.30 (18)	€1,301.80
Baha® connect (minor outpatient surgery + 35%)	€239.24 (18)	€322.98
Osia® system device (implant + sound processor)		€11,650
Sound processor	€4,730 (30)	
Baha® connect device (implant + sound processor)		€7,985
Sound processor	€4,730 (30)	

It was assumed that the management of moderate complications would require an otorhinolaryngology specialist visit and a nurse visit, while severe complications would require an otorhinolaryngology specialist visit and minor outpatient surgery. This resulted in costs of €199.66 for moderate complications and €386.67 for severe complications.

For procedures costs, clinicians agreed that implantation and explantation costs were the same. In the case of Osia® System, it was assumed that the procedure was a major outpatient surgery, €964.30, as it would require general anesthesia. The procedure for the implantation/explantation of Baha® Connect System was assumed to consist of a minor outpatient surgery, €239.24, as it would not require general anesthesia.

Reimplantation costs were assumed to be 35% higher than implantation/explantation costs, based on clinical expert inputs indicating that reimplantation procedures require approximately 35% longer operative time due to increased surgical complexity. As operating room costs are primarily driven by procedure duration and associated staff and facility time, this was considered an appropriate proxy for the additional variable cost component of reimplantation procedures. This brings the re-implantation cost to €1,301.80 for Osia® System, and €322.98 for Baha® Connect System.

### 2.7 Utilities

Since no Spanish utilities were identified during the literature review to populate this parameter, it was decided to use original model utilities, which were derived from the Health Utilities Index Mark III (HUI-3) (20). This instrument provides a more sensitive and accurate reflection of health-related quality of life in patients with hearing implants compared to other econometric instruments, as it encompasses eight domains, including hearing and speech.

- Without implant: 0.67 (21)
- With implant:
- Osia® system: +0.09 (21)
- Baha® connect system: +0.048 (22)

Severe and moderate adverse events were associated with a disutility of 0.02 (23).

## 3 Results

### 3.1 Base-case analysis

Table 3 details the base-case cost-effectiveness analysis. Over a lifetime horizon, the Osia® System incurred total costs of €24,555 per patient and yielded 15.9414 QALYs. In contrast, the Baha® Connect System had total costs of €20,968 per patient and 15.1703 QALYs. The incremental cost of the Osia® System was €3,587, with an incremental gain of 0.7711 QALYs, resulting in an ICER of €4,651 per QALY gained.

**Table 3:** Base-case cost-effectiveness results for the Spanish adaptation with a lifetime horizon from the Spanish National Health System perspective.

Intervention	Total costs per patient (€)	Total QALYs per patient	Incremental costs (€)	Incremental QALYs	ICER (€/QALY)
Baha® connect system	20,968	15.1703	Reference	Reference	Reference
Osia® system	24,555	15.9414	3,587	0.7711	4,651

Costs and QALYs are discounted at 3% annually. QALYs, quality-adjusted life years; ICER, incremental cost-effectiveness ratio.

### 3.2 Sensitivity analyses

**Figure 2:**
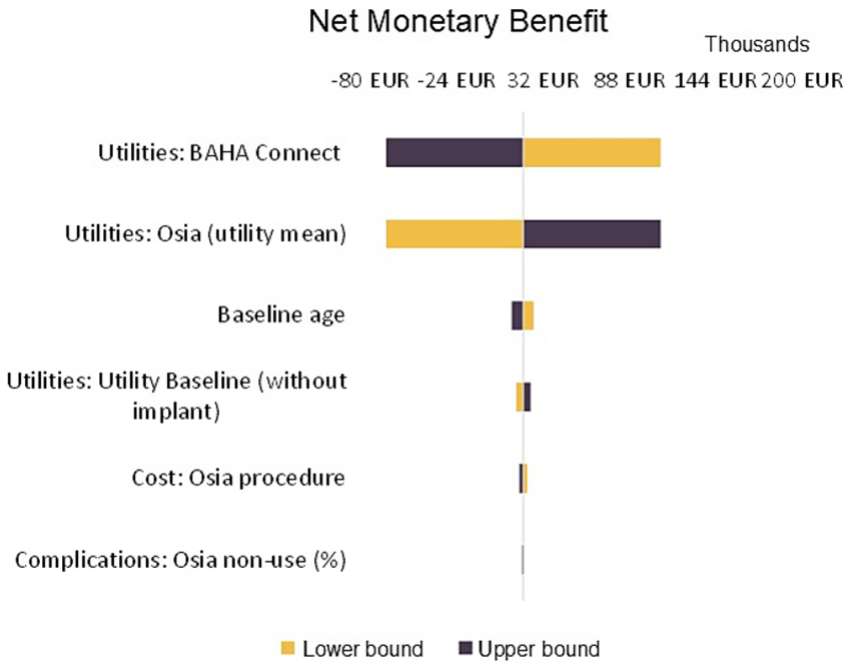
DSA tornado diagram.

The results of the one-way deterministic sensitivity analysis are summarized in the tornado diagram (Figure 2). The analysis indicated that model outcomes were most sensitive to the utility values associated with the Baha® Connect and Osia® interventions. Baseline age and the pre-implantation utility value also had a moderate impact on results. Conversely, the model presented low sensitivity to variations in the Osia® procedure cost and the device non-use rate.

The results of the PSA’s, based on 1,000 Monte Carlo simulations, are illustrated in the cost-effectiveness plane (Supplementary Figure 1). Each point represents a pair of incremental cost and incremental QALYs for the Osia® System compared to the Baha® Connect System. In total, 8% of simulations were dominant (more effective and less costly), and 62% fell below the willingness-to-pay (WTP) threshold of €30,000/QALY gained.

**Figure 3:**
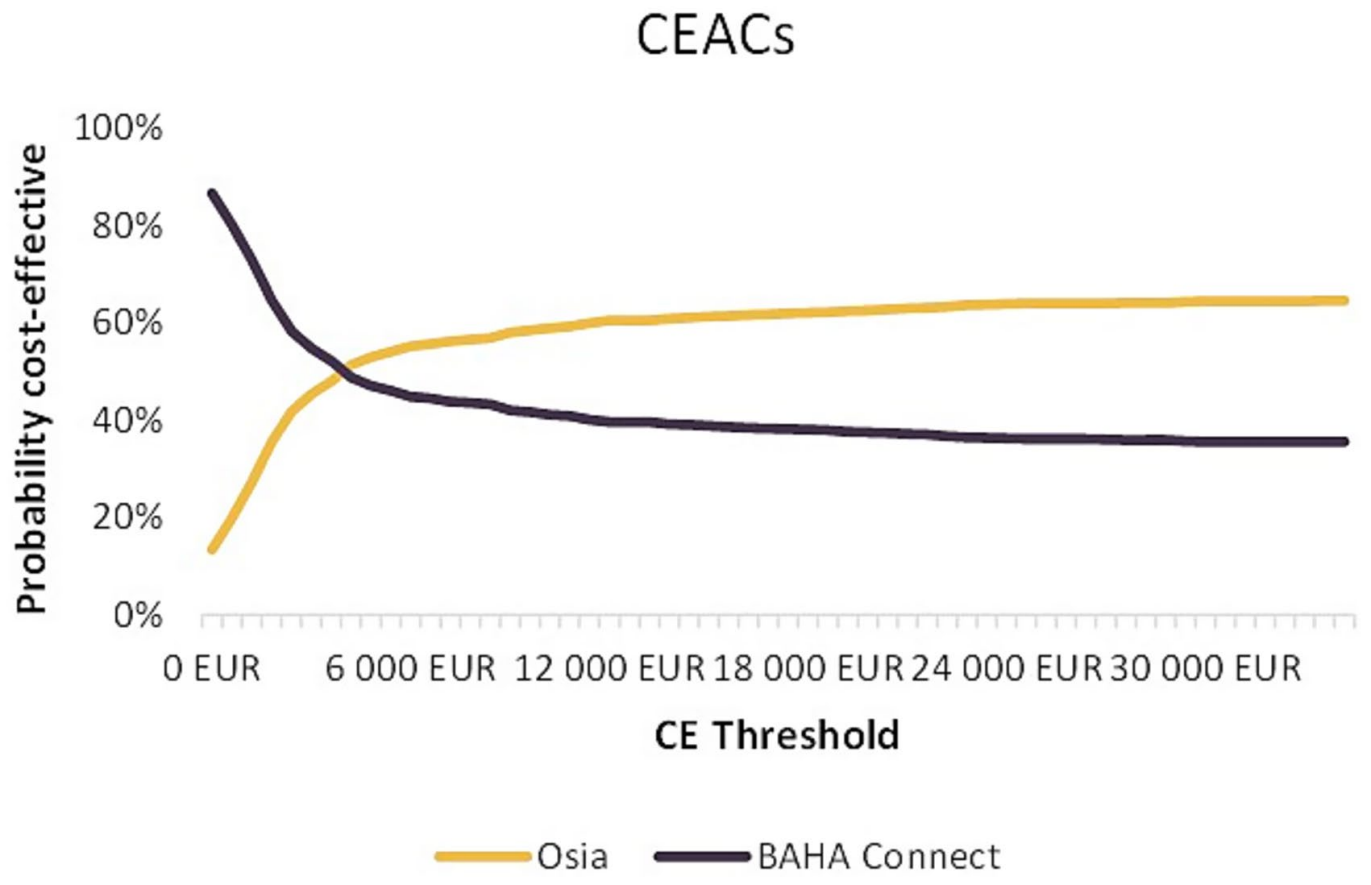
Cost-effectiveness acceptability curve (CEAC).

Figure 3 displays the cost-effectiveness acceptability curve derived from the PSA. The probability of the Osia® System being cost-effective remained above 60% across a wide range of conventional WTP thresholds. Specifically, at a threshold of €30,000/QALY, the probability of cost-effectiveness was 62%.

## 4 Discussion

This cost-utility analysis, conducted from the perspective of the SNS, suggests that the Osia® System is a cost-effective alternative to the percutaneous Baha® Connect System for adult BCHI-eligible patients in Spain. The base-case analysis yielded an ICER of €4,651/QALY gained. One-way DSA identified utility values for both interventions, baseline age, and pre-implantation utility as the most influential parameters. PSA showed that 62% of simulations fell below a WTP threshold of €30,000/QALY gained, with 8% indicating that Osia® System was a dominant strategy. At a threshold of €25,000/QALY, the probability of cost-effectiveness remained substantial, supporting the robustness of the base-case findings.

Interpreting this ICER in the Spanish context is particularly relevant given the absence of an officially defined WTP threshold. In practice, healthcare decision-makers in Spain often reference an informal range between €22,000 and €30,000 per QALY (24), and the ICER for the Osia® System lies notably below this range. At the lower bound of the threshold (€22,000/QALY), the Osia® System remains cost-effective based on the DSA. The PSA further supports this interpretation by providing a probabilistic view of cost-effectiveness, which is especially relevant in settings without a fixed WTP benchmark. The cost-effectiveness acceptability curve offers decision-makers a visual tool to assess the likelihood of cost-effectiveness across different thresholds. Together, these findings support the Osia® System as a value-based intervention aligned with the SNS resource allocation principles.

The ICER estimated in this analysis (€4,651/QALY) is notably lower than that reported in the original Swedish study (14), which found an ICER of 108,318 SEK/QALY for the Osia® System compared to percutaneous devices. When converted using an approximate exchange rate of €1 ≈ 10.9 SEK (April 2026), the Swedish ICER corresponds to around €9,937/QALY, more than twice the Spanish estimate. Interestingly, acquisition and initial procedural costs for Osia® System are somewhat higher in Spain (€11,650) compared to the Swedish procedural cost (€10,833), which would, in theory, be expected to yield a less favorable ICER in Spain if all other factors were equal. This discrepancy likely reflects differences in clinical estimates, healthcare resource utilization, cost structures, and model assumptions between the two settings. Despite these variations, the clinical parameters used in the model are considered broadly transferable due to the high degree of standardization in BCHI indication criteria and management across Europe (17). While differences in case mix or local practice patterns could influence the ICER (e.g., a younger baseline age in Spain would further increase lifetime QALY gains), the structural consistency of the clinical outcomes ensures that the Osia® System remains a cost-effective strategy within the Spanish context. Given these inherent variations, cross-country ICER comparisons should be interpreted with caution, and less emphasis should be placed on the absolute numerical differences. Importantly, both analyses consistently support the cost-effectiveness of the Osia® System within their respective healthcare contexts, reinforcing the robustness of the findings despite methodological and contextual differences.

The sensitivity analysis in the Swedish model identified baseline age, the cost of the Osia® procedure, and the mean utility gain as the key drivers of cost-effectiveness (14). Similarly, in the Spanish model, utility values, baseline age, and pre-implantation utility emerged as the most influential parameters. This alignment in sensitivity patterns suggests that the fundamental drivers of cost-effectiveness for BCHIs are consistent across different healthcare settings, despite variations in absolute cost inputs. This structural consistency further supports the robustness of the model across healthcare systems and strengthens the validity of transferring key clinical parameters between contexts.

Our findings are also consistent with other international economic evaluations. A recent Australian cost-utility study (21) reported an ICER of $29,301/QALY gained for the Osia® System compared with the Baha® Attract System, attributing this to favorable audiological outcomes and utility gains. While our analysis utilizes Baha® Connect, the Attract findings serve as a proxy, reflecting consistent incremental gains across the broader Baha® platform. Additional evidence from Netherlands found an ICER of €3,494/QALY gained for cochlear implants versus BCDs in patients with severe hearing loss (25). While these studies assess different devices and populations, they collectively support the clinical and economic value of advanced hearing technologies across healthcare systems. In contrast to comparisons involving heterogeneous or less-established devices, the present study offers a robust analysis by comparing two clinically accepted and recommended BCHI systems, thus enhancing the real-world relevance of the cost-effectiveness findings.

A major strength of this study lies in its rigorous adaptation of a well-established and previously validated Markov model to the Spanish healthcare context. This included adherence to national HTA guidelines, such as the application of a 3% discount rate for costs and outcomes, and prioritization of Spanish-specific cost inputs wherever available. In addition, the model incorporated Spanish mortality data and local validation of healthcare resource use and clinical management pathways, ensuring contextual relevance beyond cost adaptation alone. Both DSA and PSA were conducted in accordance with local methodological standards, ensuring a robust assessment of uncertainty. Emphasis on transparency in model assumptions and data sources enhances the interpretability and policy relevance of the results. In addition, the use of a pragmatic literature review ensured that parameter selection and modeling assumptions reflected the Spanish clinical environment as accurately as possible.

Nonetheless, this study presents limitations. First, in line with the original Swedish model (14), the model assumes clinical homogeneity within CHL, MHL, and SSD populations due to the lack of subgroup-specific data. Therefore, the reported ICER should be viewed as an average for the BCHI-eligible population, and the model cannot determine which of these subgroups derives the greatest clinical or economic benefit, and cost-effectiveness for each etiology separately remains uncertain. Second, the absence of head-to-head randomized controlled trials comparing the long-term outcomes of the Osia® System versus a range of percutaneous devices necessitated the synthesis of heterogeneous evidence sources, introducing uncertainty. However, this was addressed through extensive sensitivity analyses. Third, while national average costs or data from reference centers were used, regional variations in device and procedure costs across Spain were not fully captured. Moreover, the comparator was modeled as a blended representation of percutaneous devices due to the lack of device-specific clinical sources, considering the well-established clinical equivalence across BCHI systems (8). While this approach utilizes the best available evidence, it may not reflect the specific technology mix or practice patterns across Spanish autonomous communities. Finally, clinical evidence for percutaneous systems generally includes more patients and longer follow-ups compared to those for the Osia® System, reflecting their longer presence in the healthcare setting. To mitigate this, non-use rates were conservatively modeled without extrapolation beyond 2 years.

## 5 Conclusion

This study provides robust evidence that the Osia® System is a cost-effective alternative to the Baha® Connect System for adult patients with hearing loss in Spain. The economic evaluation, adapted to the Spanish healthcare context, highlights its value both in terms of clinical benefit and resource efficiency. These findings can support national decision-making processes regarding coverage, reimbursement, and guideline development for hearing implant technologies. As hearing loss continues to pose a growing public health challenge in Spain, integrating cost-effective solutions such as the Osia® System into clinical pathways may contribute to improved patient-centered outcomes and sustainable healthcare delivery.

## Data Availability

The original contributions presented in the study are included in the article/Supplementary material, further inquiries can be directed to the corresponding author.
