# Supplementary figures and images for: A cost-utility analysis of the transcutaneous Osia System® compared to the percutaneous bone-conduction hearing implant Baha® Connect for hearing loss in Spain
Source: Front Public Health. 2026 Jun 24;14:1829029. doi: 10.3389/fpubh.2026.1829029 (PMC13341937; doi:10.3389/fpubh.2026.1829029)

## Supplementary Material

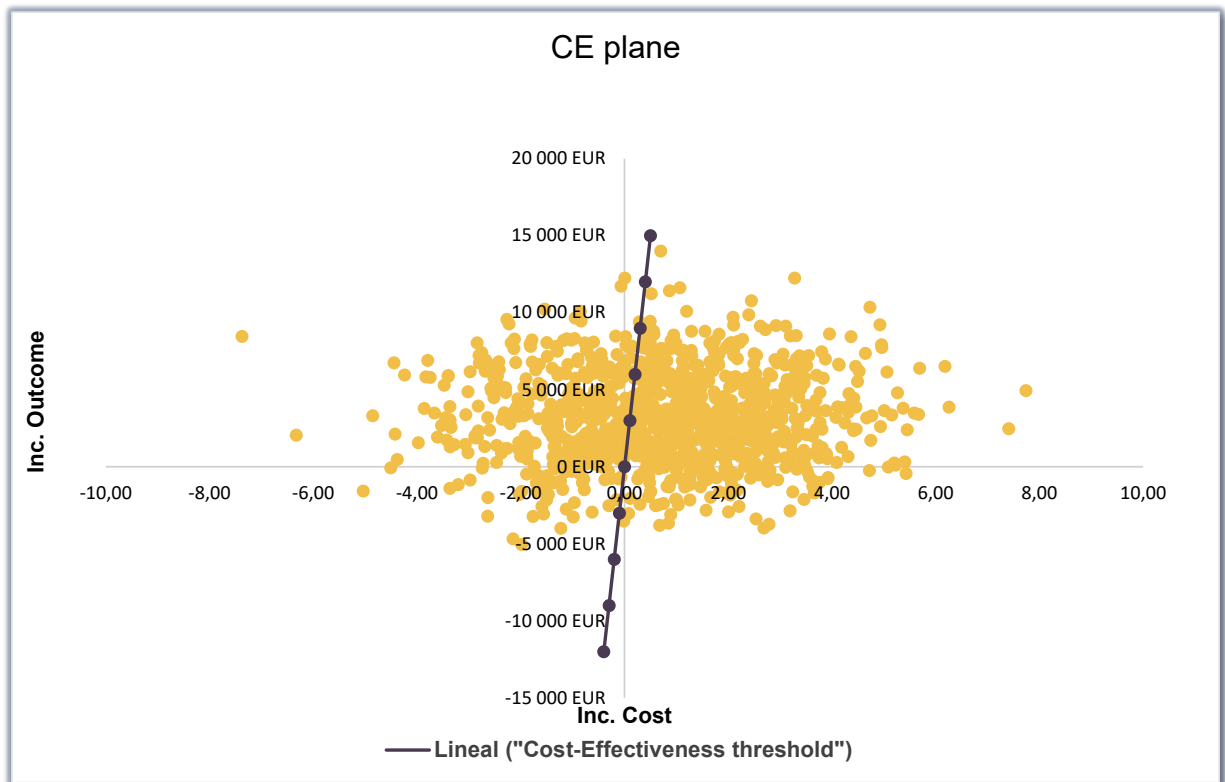

Supplementary Figure 1. Cost-effectiveness plane

Supplement: Supplementary file 1 [file Data_sheet_1.pdf]
